# Investigation of Suspected Pulmonary Embolism at Hutt Valley Hospital with CT Pulmonary Angiography: Current Practice and Opportunities for Improvement

**DOI:** 10.1155/2015/357576

**Published:** 2015-03-01

**Authors:** Nick Kennedy, Sisira Jayathissa, Paul Healy

**Affiliations:** Hutt Valley District Health Board, Lower Hutt, Wellington 5040, New Zealand

## Abstract

*Aims*. To study the use of CT pulmonary angiography (CTPA) at Hutt Hospital and investigate the use of pretest probability scoring in the assessment of patients with suspected pulmonary embolism (PE). *Methods*. We studied patients with suspected PE that underwent CTPA between January and May 2012 and collected data on demographics, use of pretest probability scoring, and use of D Dimer and compared our practice with the British Thoracic Society (BTS) guideline. *Results*. 105 patients underwent CTPA and 15% of patients had PE. 13% of patients had a Wells score prior to their scan. Wells score calculated by researchers revealed 54%, 36%, and 8% patients had low, medium, and high risk pretest probabilities and 8%, 20%, and 50% of these patients had positive scans. D Dimer was performed in 58% of patients and no patients with a negative D Dimer had a PE. *Conclusion*. The CTPA positive rate was similar to other contemporary studies but lower than previous New Zealand studies and some international guidelines. Risk stratification of suspected PE using Wells score and D Dimer was underutilised. A number of scans could have been safely avoided by using accepted guidelines reducing resources use and improving patient safety.

## 1. Introduction

Suspected PE is a common medical presentation and problem encountered amongst hospital inpatients. Due to the potential consequences, making an accurate and timely diagnosis of PE is important. As the symptoms and signs are nonspecific, laboratory investigations and imaging are required to accurately diagnose those with a PE. The speed and availability of highly sensitive multislice CTPA scanning has led to this becoming the imaging investigation of choice. As part of a clinical algorithm involving a pretest probability score and D Dimer, a CTPA can make the diagnosis of a clinically relevant PE in 98% of cases [1]. CTPAs also have the advantage of revealing other clinically significant diagnoses.

There is, however, a perception especially amongst radiologists that physicians are becoming more reliant on imaging and are requesting increasing numbers of CTPA scans. The perception that a low percentage of scans are positive raises the concern that patients are not being risk stratified. Radiologists note that well-validated measures such as Wells scores are not commonly used on radiology request forms.

Radiology is a limited resource which needs to be utilised in the most effective manner whilst maintaining patient safety. There are several good reasons to reduce the number of scans performed. Over recent years there has been an increased awareness of medical radiation and the potential effects this may have for a patient's future risk of malignancy. Medical radiation is linked to an increased risk of a number of malignancies such as lymphomas and many solid tissue cancers. Estimates of the magnitude of the problem vary with a 2007 review estimating that 1.5–2% of malignancies may be secondary to medical imaging [2]. The amount of radiation from a CTPA varies by machine, protocol, and patient characteristics such as patient size, but radiation levels between 10 and 20 mSv are considered typical [3]. This corresponds to three to five year's background radiation or the equivalent of approximately 750 chest X-rays [3]. The overall risk may be small but for certain patient groups such as the young, and in particular young females, those who are pregnant/postpartum, and patients requiring multiple scans, this risk needs to be taken into account [4]. However, for an individual patient, the risk is small; the increased number of scans performed could translate into many cases of radiation induced cancers. In addition, the intravenous contrast could result in reactions or worsening of renal function among patients with preexisting renal impairment [5].

Clinical algorithms are a well-researched and validated method of decreasing the need for CTPAs. A number of risk stratification tools exist with the Wells score being the most widely used in New Zealand. This uses the presence of clinical signs of deep vein thrombosis, absence of other diagnoses that better explain presentation, heart rate greater than 100, immobility or recent operation, previous thromboembolism, haemoptysis, and presence of underlying or recently treated cancer to class patients as either low, medium, or high probability [6]. The Wells score has been widely adopted and introduced into guidelines such as the BTS guidelines [7] as well as many New Zealand hospital guidelines [8]. In conjunction with a negative D Dimer, a low or medium risk score has been reliably shown to exclude PE [1]. We conducted this study to describe local practice and audit our compliance with BTS guidelines.

## 2. Methods

### 2.1. Setting and Subjects

Hutt Valley Hospital is a secondary hospital based in Lower Hutt Wellington, which services a population of 145,000. Its services include general medicine, cardiology, older people rehabilitation services, general surgery, plastic surgery, obstetrics, and gynaecology. This retrospective audit was completed from January 2012 to May 2012 and included all patients investigated with a CTPA for suspected PE.

### 2.2. Study Design

Patients who had a CTPA over the period were identified from radiology records. A retrospective audit was performed utilising the Concerto computer information system which includes emergency department notes, medical admission and discharge notes, and laboratory and radiology results. Written referral letters and inpatient notes were also reviewed. From these sources, information was collected on patient demographics, components of the Wells score, investigations including D Dimer, CXR, and ABG, and the CTPA scan results.

The Wells score (Table 1) is a validated clinical prediction rule which classifies patients as either low (<2), intermediate (2–6), or high (>6) risk based on the following criteria. A modified version also exists that classifies patients as PE unlikely (<4) or PE likely (≥4).

Study data was collated on an Excel spreadsheet for analysis. The notes were reviewed and data entered in a manner to ensure the Wells score was calculated prior to seeing the D Dimer or CT scan result. This process replicated the information that would be available to the doctor assessing the patient. All Wells scores were calculated by the author.

We used descriptive statistics of who ordered the scans, number of positive scans by ordering department, use of D Dimer, and percentage of patients who had Wells score calculated. Patients were stratified by Wells score and CTPA result. We calculated the utility of the D Dimer test in predicting the diagnosis of PE. Formal statistical analysis comparing the groups was not done due to small sample size. This audit was done as a quality improvement activity by the employees of the organisation and according to national ethics committee guidelines did not warrant formal ethics approval.

## 3. Results

**Figure 1:**
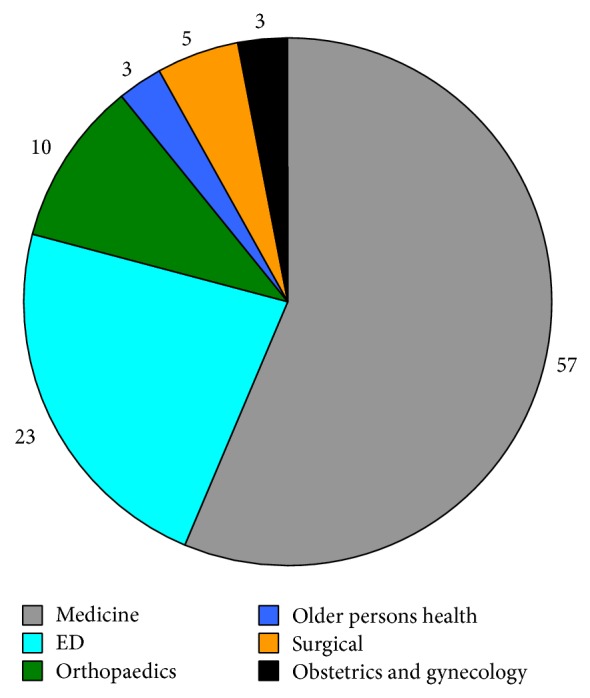
Number of CTPAs by department.

Over the 4-month period of this audit 105 CTPAs were performed. Of the 105 scans, 101 were for inpatients and all these inpatient scans were performed within 24 hours of request. The majority of the cases were performed by the general medical department with substantial numbers also performed by the emergency department and smaller numbers by other departments (Figure 1).

**Figure 2:**
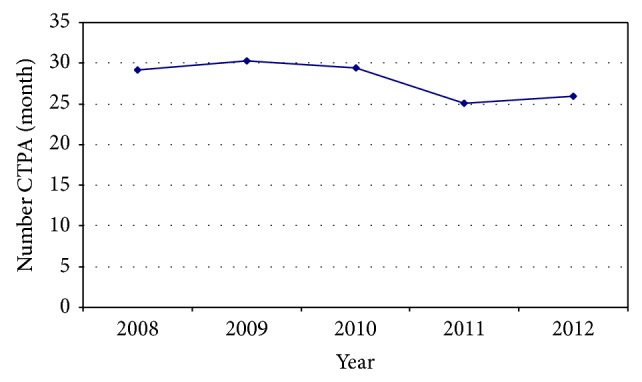
Mean CTPAs per month.

The common perception that the number of CTPAs undertaken at Hutt Hospital has been increasing does not appear to be true. Since the introduction of the PACS radiology system in mid 2008 the number of CTPAs has remained static and is perhaps slowly reducing (Figure 2).

**Figure 3:**
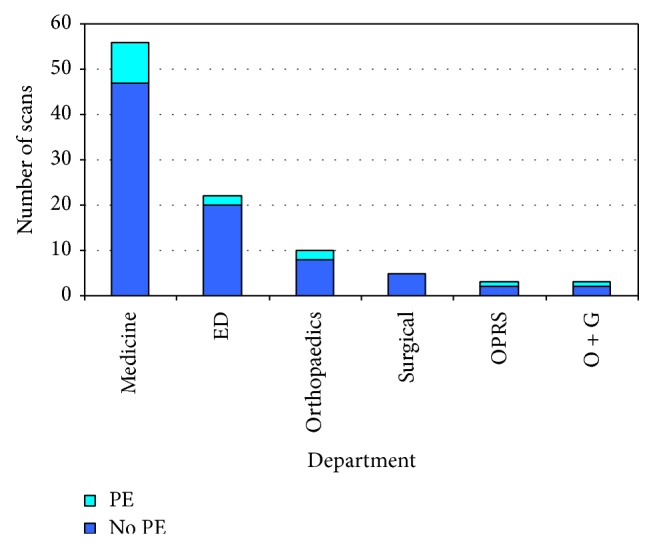
Results of scans by department.

The mean age of patients scanned was 63.7 years (range 20–98). 60% of patients were female. 18% had a history of previous thromboembolism, 11% had a history of active or recently treated cancer, and 22% had had a recent operation or significant immobility. Four of the patients were either pregnant or postpartum. 15% of scans were positive for pulmonary embolism. There was no marked variation in rate of positive scans by department (Figure 3).

In addition to the diagnosis of pulmonary emboli, a number of other clinically relevant findings were revealed. These included three malignancies (one mesothelioma and two recurrences of breast cancer), a ruptured oesophagus, omental infarct, cholecystitis, possible sarcoidosis, and pericardial and pleural effusions. A number of these findings may not have come to light with alternative investigations and many significantly altered clinical management. 16% of scans were reported to have pulmonary nodules reaching Fleischner criteria.

93% of patients received a CXR prior to CTPA. Only a third of patients received a blood gas with many of these being venous gases. A D Dimer was performed in 58% of cases. Of the D Dimers performed, 56 were positive and 5 were negative. No patients with a negative D Dimer were found to have a PE (negative predictive value 100%). The positive predictive value of D Dimer in the diagnosis of PE was 19.6%. The median D Dimer was markedly higher in those who were found to have a PE (2545) compared to those who did not have a PE (949).

12% of patients had some form of pretest probability document in the notes. Of these, 12 patients had a Wells score calculated whilst 1 patient had both a Geneva and Wells score calculated.

**Figure 4:**
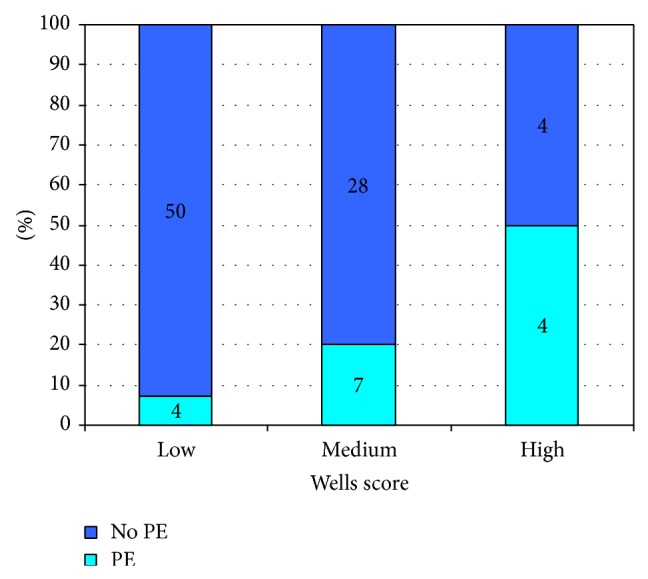
Stratification of patients via Wells score and PE status.

Of the 105 patients in the study, pretest probability was able to be calculated in 97 cases (92%). Inability to locate notes or insufficient data prevented calculation in the remaining 8%. Wells score was calculated showing 54%, 36%, and 8% of patients would have been classified as low, medium, and high risk, respectively. In these groups the rate of positive scans was 8%, 20%, and 50%, respectively (Figure 4).

No patients with a PE as their primary diagnosis died during their admission. One patient found to have a small PE as a secondary finding (to a perforated oesophagus) did however die. One patient was thrombolysed successfully. Of the 15 patients diagnosed with a PE, 10 had a workup for malignancy (8 CTs and 2 ultrasounds) while 8 had some form of thrombophilia screen. Many of these were incomplete.

## 4. Discussion

The positive yield of CTPA for PEs in the audit period was 15%. This is considerably lower than other published New Zealand studies. A similar Christchurch study in 2006 had a CTPA positive rate of 31% [9] while a 2003/2004 study from Hamilton of 523 patients with a moderate or high pretest probability score had a CTPA positive rate of 20.1% [10]. A recently published study from Timaru had a similar CTPA positive rate to our study of 14% [11].

Recent overseas studies [12, 13] have shown similar rates of PE to this audit ranging from 10% to 20%. Some studies suggest that the CTPA positive rate is falling with time reflecting overall increased use of medical imaging [14]. The Royal College of Radiologists (UK) [15] suggests 15.4–37.4% is an acceptable CTPA positive rate.

The use of validated pretests was significantly underutilised with just 12% of patients having either a Wells or Geneva score performed. While this number is low, it is higher than the Christchurch study [9] where just 4% was performed.

A D Dimer was performed in only 58% of cases. This represents a significant underutilisation of what in the correct setting is a useful test to reliably and safely exclude PE and remove the need for CTPA. In this audit, no cases with a negative D Dimer were found to have a PE. This is in keeping with the well-established high sensitivity of this test [16]. The BTS guidelines state that “A negative D Dimer test reliably excludes PE in patients with low or intermediate clinical probability; such patients do not require imaging for VTE.” In this audit four patients with a low or medium risk score and a negative D Dimer underwent a CTPA. All of these scans were negative.

33% of patients in this study fell into the low or intermediate risk categories yet did not have a D Dimer performed. Following the above BTS recommendation a negative D Dimer in any of these patients would have safely removed the need for a CTPA. While it is not possible to know the exact number of scans this could have avoided, it is a significant deviation from best practice. An estimate could be made using data from large studies such as the Christopher study [1] where 32% of patients being assessed for possible PE had a negative D Dimer. If similar rates were seen in our patient population there could have been 10 further scans safely avoided.

One way to increase compliance with BTS guidelines is to necessitate risk stratification and D Dimer prior to a CTPA. Studies doing this have showed a decrease in the number of scans requested and subsequently a higher percentage of positive scans [17]. The main barrier to successfully introducing such systems is clinician compliance [17].

This study has a number of limitations due to its design as an audit. The numbers are relatively small and conclusions can only be applied to Hutt Valley Hospital. There might be a conceptual problem with calculating pretest probability scores retrospectively based on clinical notes [9] although audit procedures were designed to ensure that all information used in calculating a Wells score by the researcher was available to the treating clinician. Where a Wells score was documented in the notes, this was used over that calculated by the author. In these instances scores were very similar.

54% of the patients had low Wells score and 8% of them had confirmed PE. On the other hand 8% of patients had high Wells score and 50% of them had PE. These results were similar to derivation and validation set by le Gal and others [18]. In the present study we did not calculate the predictability of combined D Dimer and Wells score as the study was aimed at examining the utilisation of these tests in patients who had CTPA. Most studies looking at validity of the Wells score and D dimer in diagnosis of PE, and calculating ROC and predictability of tests, conducted these tests according to a preplanned protocol. Wells score has a reasonable clinical utility with the AUC of the continuous prediction rules around 79% [19].

The complexities of clinical decision making can be difficult to fully document, so finer points may have been lost though a review of clinical notes. As an example, the clinician's decision to request a CTPA may have only been partially based on a need to exclude a PE. Other diagnosis may have been also considered and appropriately investigated with CT scanning. In this study a number of other pathologies that may not have otherwise come to light were identified. Many of these significantly altered patient management. This finding is in keeping with other studies where a high frequency of other clinically relevant pathology is diagnosed on CTPA [11, 14]. 16% of scans had nodules requiring further investigation or followup. Some of these may lead to early diagnosis of malignancy. We have not specifically looked at the clinical utility of finding alternative diagnoses in this study.

Overall this study showed that while the CTPA positive scan rate was similar to other contemporary studies [12, 13] it was lower than previous New Zealand studies [9, 10] and international recommendations [15]. This suggests we are performing too many scans. Stratification of suspected PE was rarely performed and D Dimer was significantly underutilised. This is likely to have led to a number of CT scans being performed which could safely have been avoided. We estimate this to be 15% of scans in this series. If risk stratification and D Dimer had been routinely performed our rate of CTPA positive scan would have fallen within The Royal College of Radiologists (UK) recommendation [15].

## 5. Conclusions

By risk stratifying patients, using D Dimer appropriately and following BTS guidelines, physicians can avoid unnecessary CTPA scans while safely investigating an important medical problem. This would help reduce the burden on radiology services enabling them to be more efficient, decrease waiting time for other radiology investigations, and reduce patient exposure to unnecessary radiation.

Audits of this nature improve clinical practice by reducing waste and patient harm and we would encourage other New Zealand hospitals to conduct similar studies to critically look at their practice and make improvements. Repeating such audits at regular intervals would help to determine the impact of quality improvement activities on clinical practice.

## Figures and Tables

**Table 1:** Wells score.

Clinical symptoms of DVT	3 points
Other diagnosis less likely than PE	3 points
Heart rate > 100/min	1.5 points
Immobilization ≥ 3 days or surgery in previous four weeks	1 .5 points
Previous DVT or PE	1.5 points
Haemoptysis	1 point
Malignancy	1 point
